# Flexible Waterproof Piezoresistive Pressure Sensors with Wide Linear Working Range Based on Conductive Fabrics

**DOI:** 10.1007/s40820-020-00498-y

**Published:** 2020-08-08

**Authors:** Hongcheng Xu, Libo Gao, Yuejiao Wang, Ke Cao, Xinkang Hu, Liang Wang, Meng Mu, Min Liu, Haiyan Zhang, Weidong Wang, Yang Lu

**Affiliations:** 1 School of Mechano-Electronic Engineering, Xidian University, Xi’an, 710071 People’s Republic of China; 2 CityU-Xidian Joint Laboratory of Micro/Nano-Manufacturing, Xi’an, 710071 People’s Republic of China; 3 Department of Mechanical Engineering, City University of Hong Kong, Kowloon, 999077 Hong Kong SAR People’s Republic of China; 4 Nano-Manufacturing Laboratory (NML), Shenzhen Research Institute of City University of Hong Kong, Shenzhen, 518057 People’s Republic of China; 5 Micro-/Nano-technology Research Center, State Key Laboratory for Manufacturing Systems Engineering, Xi’an Jiaotong University, Xi’an, 710049 People’s Republic of China

**Keywords:** Flexible sensor, Piezoresistive, Graphite flakes, Laser engraving, Silver fabrics

## Abstract

**Highlights:**

- The laser-engraved method was introduced to fabricate the electrode for the sensor.
- The sensor showed a wide linear working range, superior sensitivity, and fast response time and also exhibited excellent viability in a wet situation.
- Wireless integrated network sensors successfully monitored the health states.

**Abstract:**

Developing flexible sensors with high working performance holds intense interest for diverse applications in leveraging the Internet-of-things (IoT) infrastructures. For flexible piezoresistive sensors, traditionally most efforts are focused on tailoring the sensing materials to enhance the contact resistance variation for improving the sensitivity and working range, and it, however, remains challenging to simultaneously achieve flexible sensor with a linear working range over a high-pressure region (> 100 kPa) and keep a reliable sensitivity. Herein, we devised a laser-engraved silver-coated fabric as “soft” sensor electrode material to markedly advance the flexible sensor’s linear working range to a level of 800 kPa with a high sensitivity of 6.4 kPa^−1^ yet a fast response time of only 4 ms as well as long-time durability, which was rarely reported before. The integrated sensor successfully routed the wireless signal of pulse rate to the portable smartphone, further demonstrating its potential as a reliable electronic. Along with the rationally building the electrode instead of merely focusing on sensing materials capable of significantly improving the sensor’s performance, we expect that this design concept and sensor system could potentially pave the way for developing more advanced wearable electronics in the future.
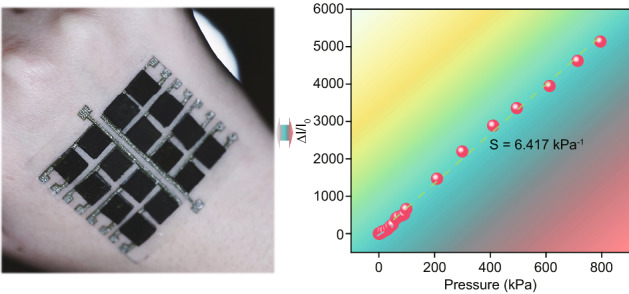

**Electronic supplementary material:**

The online version of this article (10.1007/s40820-020-00498-y) contains supplementary material, which is available to authorized users.

## Introduction

Flexible pressure sensor, especially the piezoresistive sensor, is widely employed in electronic skin [1–4], healthcare monitoring [5–8], and human–machine interactions (HMI) [9–11]. To expand the feasibility of the piezoresistive sensor for diversified practical applications, they should have a linear pressure-sensing capability within large dynamic sensing ranges to constantly maintain their high sensitivity from low-pressure (< 10 kPa) to high-pressure region (> 100 kPa, even near 1 MPa) [12–14]. So far, however, this is still a great challenging task for this kind of pressure sensor [15].

In parallel, although so far extensive new approaches that have been developed to address the balance between the high sensitivity and wide working range, not all are fully effective, and many necessitated tedious strategies for practical application [16]. In principle, the piezoresistive effect mainly derived from the contact resistance changes between the sensing material and the electrode or the bulk resistance within the sensing material [17]. Compared to the latter, the contact resistance contributes to the most conductivity variation under the same applied pressure [18]. The reason is that a small increase in the contact area between the electrode and the active material leads to a significant decrease in the contact resistance. Inspired by this, constructing ordered rough architectures (e.g., the planar, microdome, micropyramid, and the micropillar structure) on the sensing materials were extensively studied to increase the contact variation in improving the sensitivity and working range [12, 19–22]. Fewer reports, however, including our previous studies, achieved a wide working range of over 100 kPa with superior sensitivity were studied through the development of the sensing materials [23–25]. Regarding this, more deep experimental research on the modification of electrode structure instead of merely the sensing materials is needed to attempt to overcome this hurdle and excavate more potential to improve both the working range and sensitivity. As a typical demonstration, our recent work used an insulating bio-derived porous layer as an insulating spacer between the sensing and electrode materials achieved a practical and energy-conservation approach [26].

In this study, the laser-engraved soft silver-coated fabric (SF) with ordered textures was regarded as the sensor’s integrated electrode, while the graphite flakes (GFs)-modified non-woven fabrics (G-nWF) were served as sensing materials. Benefited from the unique structure of the electrode and sensing materials’ random rough surface, we surprisingly push the flexible sensor’s linear working range to a new level of 800 kPa with a high sensitivity of 6.4 kPa^−1^, a fast response time of only 4 ms, and long-time durability. Our sensor device also exhibited improved viability in the wet environment due to its rationally designed microscale polymeric forests. Finally, a wireless signal wearable sensor device was successfully demonstrated to send the pulse rate information to the portable smartphone. The methodology and platform offered a powerful tool for designing and creating advanced flexible sensor with a wide working range and superior sensitivity through the “electrode” aspect and which also could potentially be used for other advanced electronics.

## Experimental Section

### Preparation of the PDMS Micropillar Film (PDMS MPF)

Prior to the preparation of the PDMS MPF, the PDMS solution was firstly prepared by mixing a base (Dow Corning, Sylgard 184 A) with a curing agent (Dow Corning, Sylgard 184 B) with a weight mixing ratio of 10:1. The stainless steel mold with cylinder pores (200 µm in diameter) was initially manufactured carefully by the laser cutting machine. Then, a certain amount of CNT solution (5 mg mL^−1^ in isopropyl alcohol (IPA)) was sprayed onto the above stainless steel foil with 1 mL cm^−2^. After the CNT solution was fully evaporated at 70 °C for 30 min, the prepared PDMS solution was poured into the mold and together put into the vacuum-drying oven to sufficiently remove the gas bubbles with the subsequence of heating at 70 °C for 1 h. Finally, the MPF covered with CNT was carefully peeled off from the substrate.

### Assembly of the Flexible Sensor

The flexible sensor mainly composed of the laser-carved integrated electrode and sensing materials. The SF (purchased from Dongguan Fenda Optoelectronics Co. LTD) was firstly mounted onto the PI/PET film (100 µm in thickness) tightly. And then, a laser etching system (maximum power of 7 W) with a wavelength of 405 nm was employed to etching the unused part, followed by using a tweezer to remove the residual SF to assist the formation of the designed soft electrode panel. The laser speed and power were set to be 1500 mm s^−1^ and 28%, respectively. The spacing distance both was set to be 800 µm, which was the optimal size can be reached for this patterning method. For the preparation of the sensing materials, the non-woven fabrics were sequentially treated with alcohol and deionized (DI) water with the following of drying the substrate at ambient temperature. And then, the prepared sample was made conductive by directly immersing into the graphene solution (2 mg mL^−1^) according to our previous research [26]. After assembling the PDMS MPF, G-nWF, and flexible SF electrode together, the self-cleaning flexible sensor was successfully fabricated.

### Phase and Structural Characterization

The sample morphology was observed via a scanning electron microscope (SEM, FEI-Quanta™ 450 FEG). The composition and crystalline structure were analyzed on transmission electron microscopy (TEM, JEOLJEM-2100F).

### Micro-mechanical Testing of GFs

The micro-mechanical tests were conducted inside a JEOL 2100 TEM using a Nanofactory TEM–scanning tunneling microscopy (TEM-STM) holder. GFs were glued to a 200-mesh copper half-grid and then glued to a gold rod using silver-conductive epoxy (Ted pella, inc) on the one side of the holder. On the other side, a tungsten needle was inserted in the holder for in situ manipulation of individual GFs.

### Electro-mechanical Measurement of the Sensor

Compression or tensile tests were applied by a universal mechanical testing machine (Zhi Qu, ZQ-990B). Corresponding resistance or current change of the sensor was directly recorded by the source meter (Keithley 2400) and electrochemical workstation (Chenhua, CHI760e) with two-electrode mode.

### Contact Angle Measurements

The water contact angle on the substrate was tested by the machine (MC-100, Changzhou Sanfeng), and it was calculated by the corresponding software. At least five tests were repeated for each sample.

## Results and Discussion

### Device Design and Working Principle

**Fig. 1:**
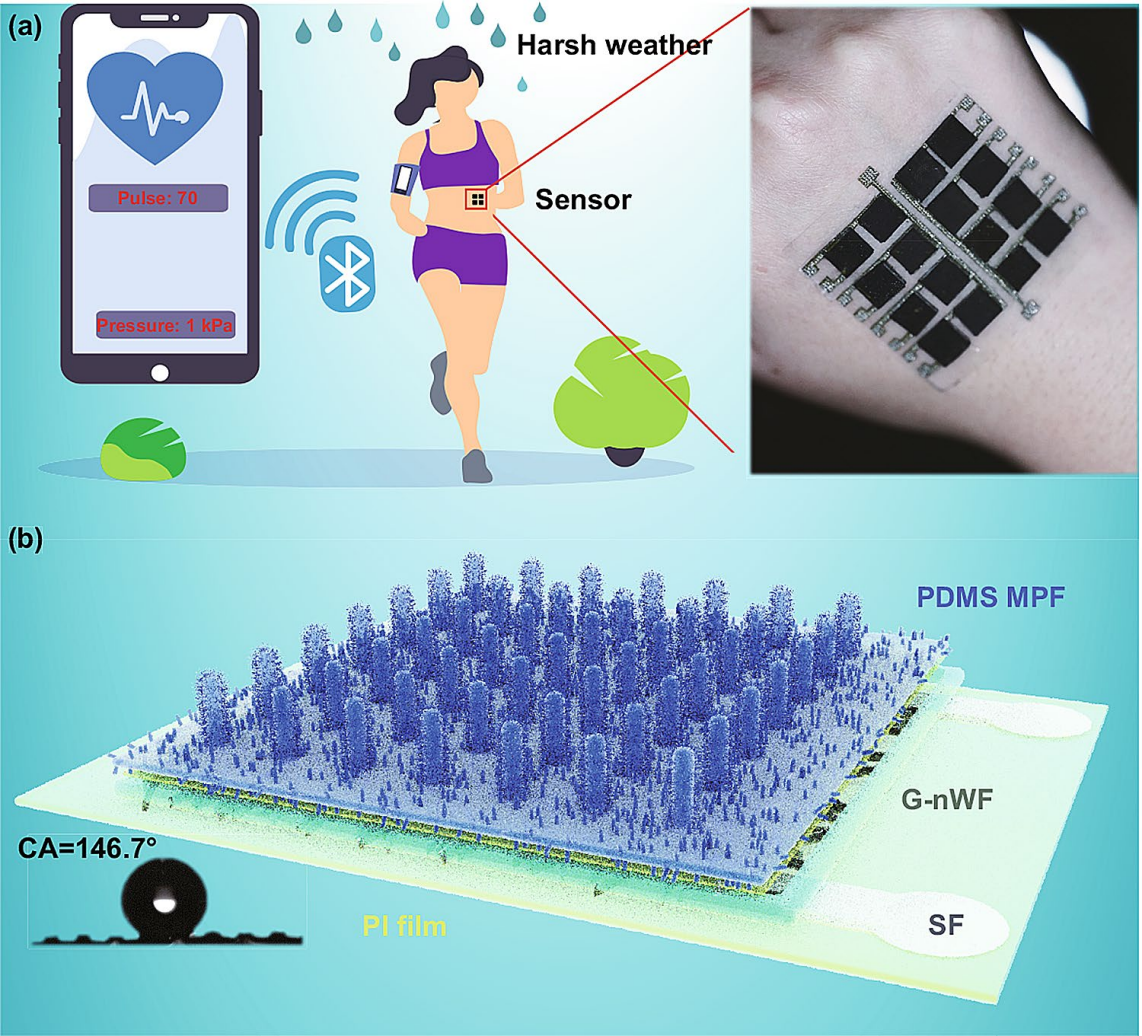
Schematic illustration of the flexible sensor. **a** Wearable sensor capable of successfully monitoring the pulse rate and sending the signal to the smartphone via the Bluetooth and the digital optical images of the waterproof sensor mounted onto our hand. **b** Schematic illustration of the structure of the sensor and its waterproof properties

The sophisticated wearable sensor typically covered the following crucial components, referred to as “wear-sense-communicate-analyze-interpret-decide” [27]. In our design concept, the wearable requirements must be first met, so all the electrode and sensing materials, as well as packing materials in the sensor device, are intrinsically soft and biologically compatible to cater for the wearable ability (Fig. 1a). Then the figure of merits for wearable sensors, including sensitivity, working range, or dynamic characteristics, should be carefully considered. Given that extensive reports have focused on the improvement of sensitivity, we thus primarily aimed at pushing the linear working range to a new horizon by using the soft SF as the integrated electrode for the first time in the flexible sensor (Fig. 1b), greatly expanding their applications of the flexible sensor compared to the traditional silicon-based micro-electromechanical systems (MEMS) device. Also, a promisingly large-scale way using the laser-engraved stainless steel foil as the template for casting a waterproof PDMS micropillar film (MPF) grafted with carbon nanotube (CNT) (water contact angle (CA) is approaching 150º) was employed to further improve its viability in harsh environment such as the raining (Fig. 1a). Once these abilities are thoroughly evaluated, a Bluetooth served as a wireless signal device was successfully integrated to achieve the “communicate-analyze-interpret-decide” function to send the detected signal, i.e., pulse rate or applied pressure to the portable smartphone, thereby bringing the flexible sensor from laboratory to the real world.

### Device Design and Working Principle

**Fig. 2:**
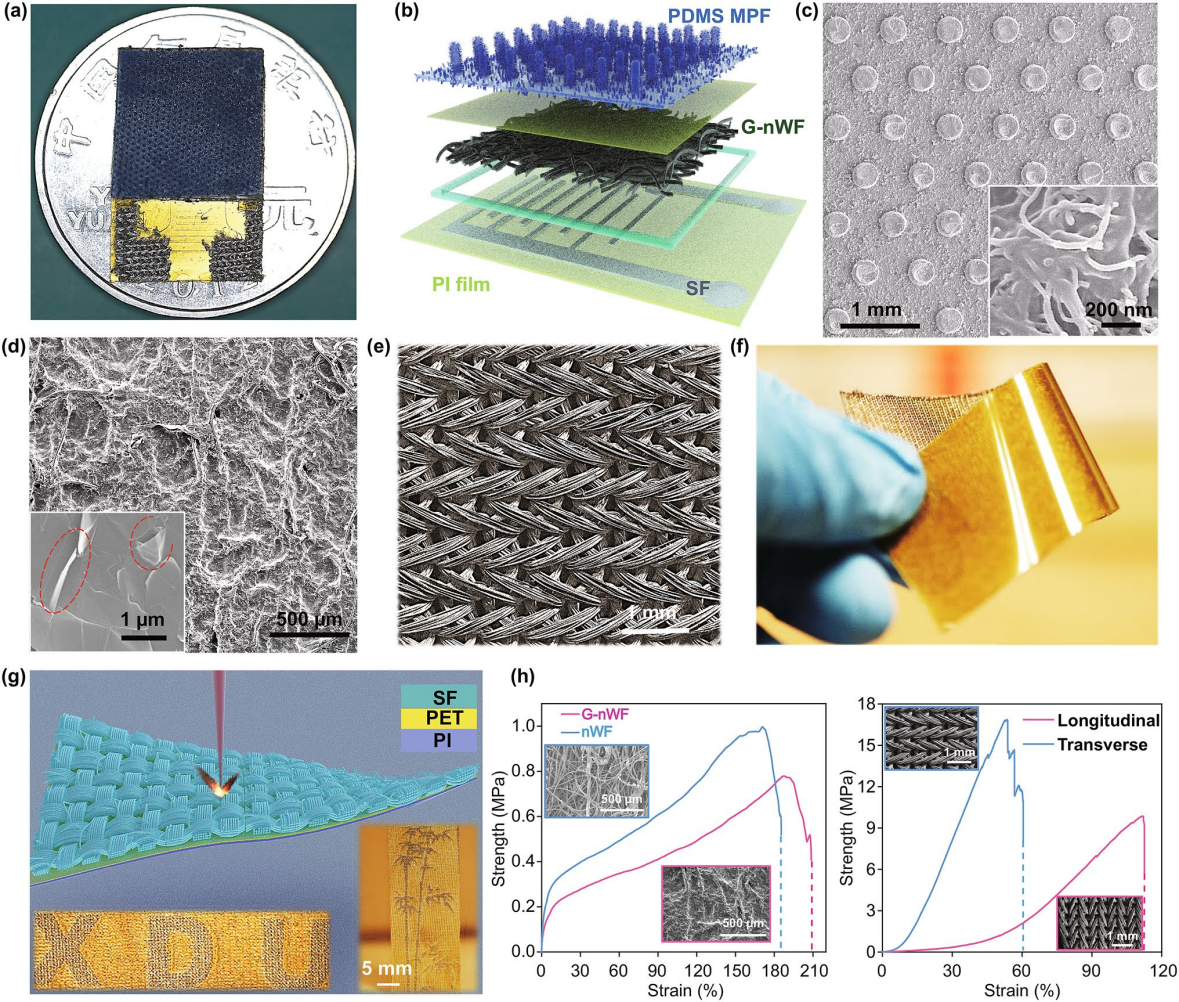
Structural characterization of the flexible sensor. **a** Digital optical image of the sensor unit on a coin. **b** Detailed structural information of the sensor. **c** SEM image and corresponding large view of the PDMS MPF. **d** SEM image and corresponding large view of the G-nWF. **e** SEM image of the SF with an ordered pattern. **f** Digital optical image of the ultra-flexible SF mounted on PI film. **g** Schematic illustration of laser engraving of the SF. Inset showed various patterns that could be readily designed and fabricated, such as the “bamboo” and “XDU.” **h** Tensile test of the G-nWF sensing materials and SF electrode, demonstrating the high flexibility

The fabricated device (Fig. 2a) mainly composed of the PDMS MPF, G-nWF, and SF electrode mounted on the PI/PET substrate (Fig. 2b). As described above, the PDMS pillar with a diameter size of 100-µm grafting CNT whiskers was successfully cast from the mold using the laser-engraved method (Fig. S1), which served as the protective armor for the device (Fig. 2c). For piezoresistive sensing materials, research has pointed to the importance of considering both conductivity and rough surface to improve its sensitivity and working range significantly [17]. In the present work, a simple yet efficient “dip-coating” method was used to fabricate such G-nWF, as shown in Fig. 2d. Hierarchical micro- and nano-sized non-flat surface, which was mainly induced by the texture of pristine nWF (Fig. S2), was beneficial to increasing the contacting area when subjected to pressure, thereby improving sensitivity and linear working range. Previously, typical electrode materials in sensors primarily focused on the gold, carbon, or silver-based materials fabricated using traditional lithography, screen printing as well as 3D printing method. However, few electrode structures comprising of highly uniform hierarchical architectures were explored to improve its performance further. In this work, a hierarchical was rationally employed (Figs. 2e and S5), which is directly derived from the soft composite SF using a laser-engraved method (Figs. 2f**–**g and S3). To gain more insight of the SF change under the illumination of the laser beam, detailed SF morphology features are shown in Figs. S5 and S6. It can be obviously observe that the SF was etched relatively smooth without any clear mechanical deformation such as curling by heating or destructive fracture. The as-obtained SF electrode exhibited a superior conductivity of 0.05 Ω cm^−2^ while retaining its original performance under large deformation (Fig. S7). More importantly, this method can be easily exploited to manufacture other interesting patterns (Fig. S8). Note that since the mechanical stretchability is essential for the flexible electronics to achieve conforming deformation, the tensile performance of G-nWF and SF electrode was therefore quantitatively investigated as shown in Fig. 2h. The G-nWF showed an inferior fracture strength of 0.8 MPa yet large tensile strain compared to the original nWF, which possibly is because the GFs significantly make the fiber aggregated together seriously and thus cannot fully exert its synergistic effect, as demonstrated by the SEM observation in the inset. The SF revealed different mechanical properties along the longitudinal and transverse direction but both excellent tensile straining of over 60%, demonstrating its practical mechanical feasibility in our flexible sensor.

### Device Characterization

**Fig. 3:**
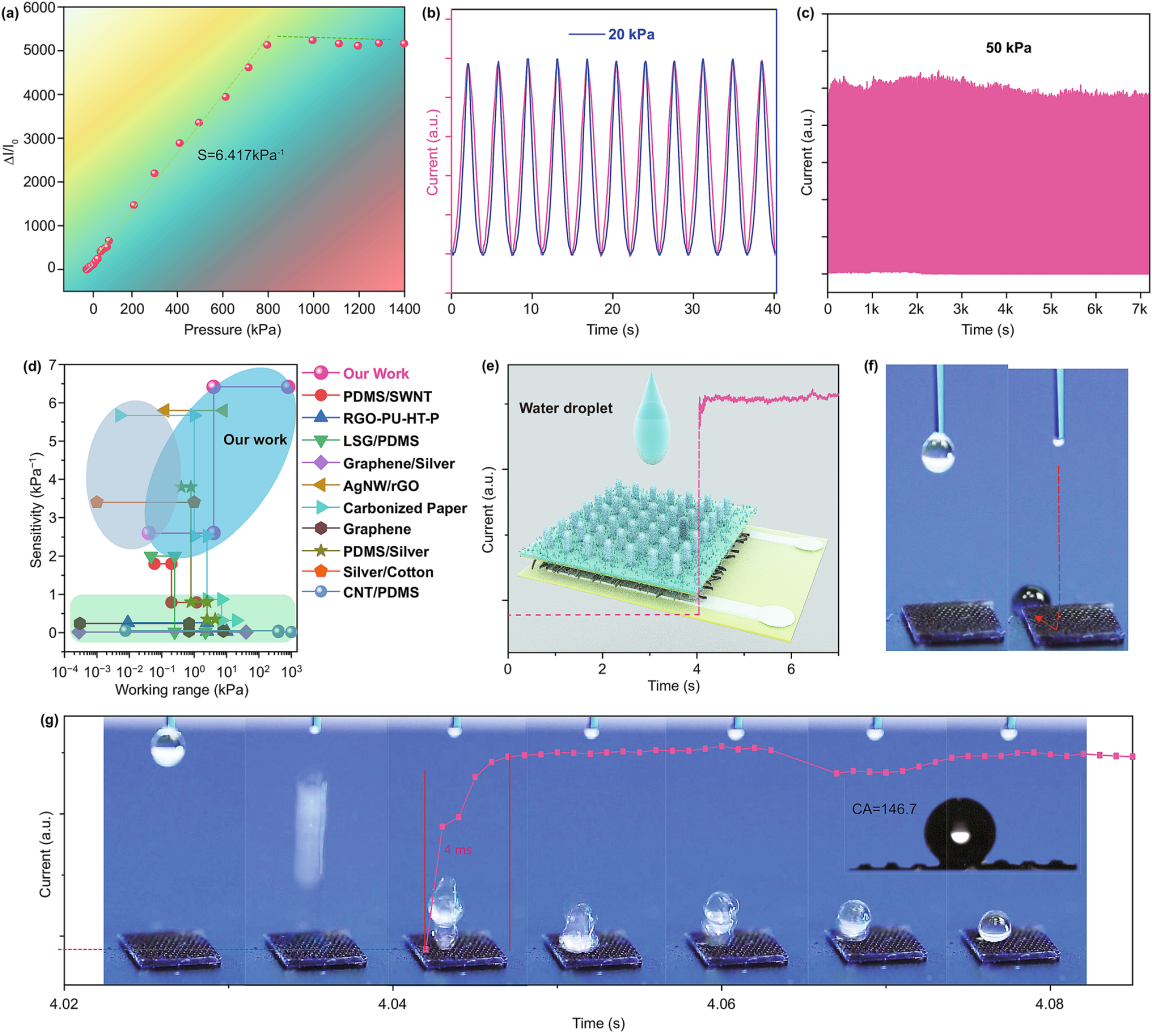
The performance of the flexible sensor. **a** Relative current variation as a function of time for various applied stress from 0 to 1400 kPa. **b** The current signal has an excellent match with corresponding applied stress. **c** Durability test of the sensor under an applied pressure of 50 kPa for 7000 s. **d** Comparison of the sensitivity and pressure range of our sensor with the previously reported sensor. **e** The response of the flexible sensor when subjected to a dropping of water. **f** The water was rapidly removed by the hydrophobic surface (see Movie S1). **g** Our sensor can easily detect the bouncing of the water drop and showed a fast response time of only 4 ms

Pressure sensors should be typically evaluated on the following items, i.e., sensitivity, working range, hysteresis, response time, and stability [17]. The sensitivity of the sensor is a critical parameter for evaluating device performance. It is typically defined as *S *= (Δ*I*/*I*_0_)/Δ*P*, where Δ*I* represents the current change before and after applying pressure, *I*_0_ represents the initial current when no pressure is applied, Δ*P* is the amount of pressure change from *I*_0_ to *I*. As shown in Figs. 3a and S10, the Δ*I*/*I*_0_ as a function of pressure is plotted. The results show a superior sensitivity of 6.417 kPa^−1^ and desired linear relation between the current change and pressure from 1.2 to 1.4 MPa, achieving an excellent balance between the sensitivity and working range. The relative stable linearity of the sensor’s current variation over 800 kPa shows the viability in extreme press loading of our device. Even below 1.2 kPa, the sensor still showed reliable sensitivity of 1.1 kPa^−1^ and the pressure resolution of the sensor is ~ 14 Pa. To our scientific knowledge, only a few reports on such a linear wide working range of the flexible sensors can be found. Under a dynamic pressure of 20 kPa, the sensor remains nearly negligible hysteresis, as shown in Fig. 3b. A pressure of 50 kPa was applied to the sensor for 7000 s to test its stability. However, no obvious decay was observed during this process, demonstrating its potential in practical application (Fig. 3c). Additionally, two higher pressures of 400 kPa and 1000 kPa were applied for 3500 s and the current response of 1000 kPa is nearly twice that of 400 kPa, exhibiting the excellent linearity as well as a fast response time of only 36 ms (Fig. S10). This superior performance not only derived from the mechanical stability, but also from the superior robust conductivity of the silver layer of the SF. Note that the most significant merit of our sensor is the rational combination of the extended working range and superior high sensitivity, which is higher than other reports with similar sensing materials (Fig. 3d) [28–37]. Furthermore, fast response time is imperative for the sensor to process the input data. As shown in Fig. 3e, a drop of water (~ 0.3 mL) fell on the surface of the sensor to test its response time and self-cleaning performances. The water would quickly be removed by the sensor due to its hydrophobic property (CA is 146.7° and roll-off angle is less than 8°) of the PDMS pillar buildings, demonstrating its application in wet conditions (Figs. 3f, S11, S12, and Movie S1). Additionally, the sensor showed an excellent response time of ~ 4 ms, and it even can detect the droplet bouncing on the superhydrophobic surfaces (Fig. 3g), thus offering a useful tool for the bouncing testing of the water droplet, in addition to a high-speed camera. We also investigated the sensor resistance changes versus different relative humidity from 20 to 90% inside the practical environment (Fig. S13). It can be found the sensor still can work at the highest humidity environment, demonstrating the sensor’s superior viability.

**Fig. 4:**
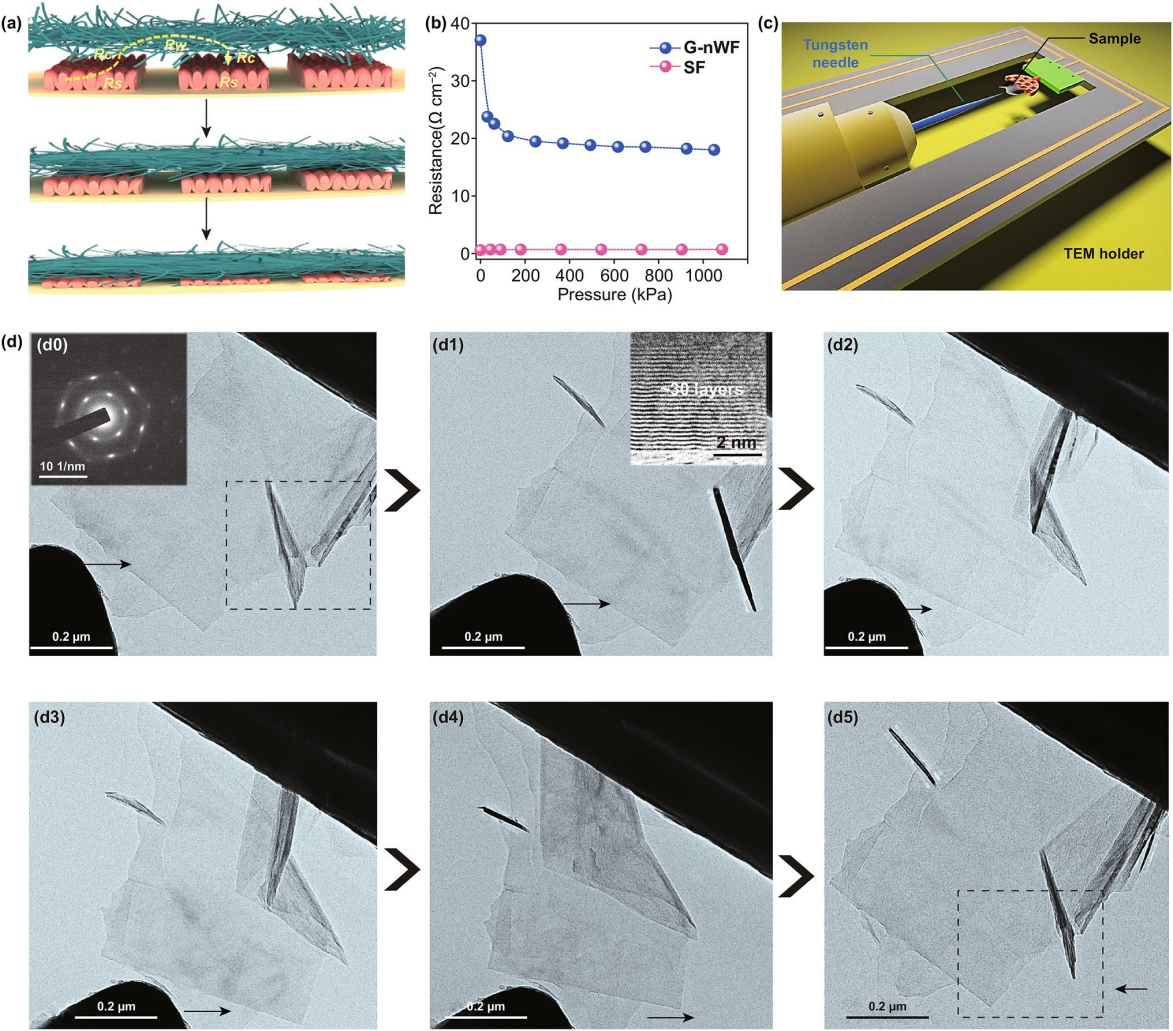
Schematic illustration of the working mechanism of the sensor. **a** The proposed operating mechanism of the sensor. **b** Resistance changes of the G-nWF and SF. **c** In situ TEM micro-mechanical device for the GFs. **d** In situ micro-mechanical characterization of GFs inside TEM, demonstrating its superior mechanical performances

To elucidate the sensing mechanism of the sensor, the corresponding illustration is depicted in Fig. 4a. The resistance is primarily consisting of the resistance change of the SF (*Rs*), the G-nWF (*Rw*), and the contact resistance (*Rc*). Initially, the SF and G-nWF would undergo mechanical deformation due to the intrinsic soft properties under the applied pressure, which possibly leads to the resistance change of the *Rs*, *Rw*, and *Rc.* However, as the quantitative analysis shown in Fig. 4b, there is almost no noticeable change of the *Rs* and only a slight variation of *Rw* during the whole applied pressure varied from 0 to 1 MPa. This confirmed that the resistance variation is mainly derived from the contact resistance but not the intrinsic bulk resistance of SF or G-nWF. Further, considering the hierarchical and unique pattern of the electrode materials (Figs. 2e and S5), the contact resistance *Rc* is primarily induced by the increased contacting area between the G-nWF and SF as a simple illustration in Fig. S14. To gain more insight, we made a comparison of our sensor with others’ work which also used the same integrated electrode and carbon-based soft sensing materials to find the deep sensing mechanism. Firstly, Chen et al. [28] and Zhan et al. [38] both used the same soft carbon sensing materials (also same with ours) yet a planar smooth integrated electrode (differ with ours) to engineer the sensor. However, the sensor only can work below 20 kPa with a reliable sensitivity. Therefore, we deduced that the modification of the electrode materials is critical for improving the sensor’s performance. This is further confirmed by Zhang et al.’s work [12]. They patterned the rough integrated electrode and also used the soft carbon sensing materials, extending the linear working range up to 200 kPa. On the basis of these results we concluded that the wide linear working range of our sensor mainly derives from the ordered structure and soft property of the electrode materials given that a nearly same sensing materials were employed in these integrated electrode-based piezoresistive sensors. Additionally, as the core sensing materials, the GFs’ mechanical properties play an imperative role in the sensor’s fast response time and long-time durability. As shown in Fig. 4c, d, the GFs are mainly consisted of 30 layers of graphene sheets (insets in Fig. 4d0 and d1). Even suffered from a serious bending (d0–d5), the GFs still can retain original state without obvious decay as demonstrated by the in situ transmission electron microscopy (TEM) nano-mechanical test. We repeated this bending process for several times, the GFs showed an excellent recoverability and flexibility (Movie S3). This also can explain why the sensor owns fast response time and superior long-time durability in addition to the excellent mechanical performance of the SF and G-nWF.

### Practical Application

**Fig. 5:**
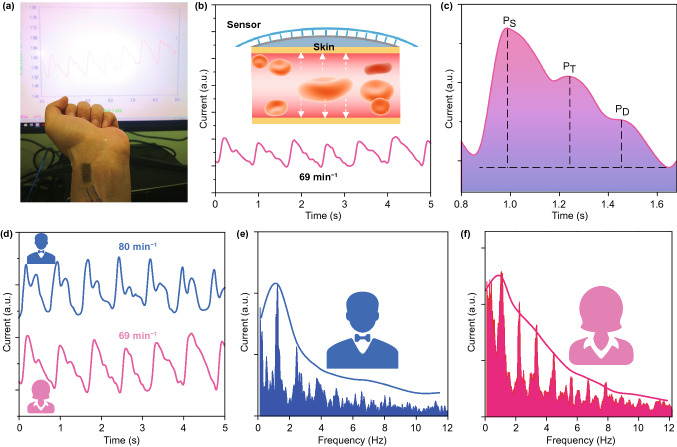
Pulse rate monitoring of the flexible sensor. **a** The flexible sensor was well mounted onto our body to monitor the pulse rate. **b**-**c** Principle and practical application of the sensor on successfully detecting the pulse rate. **d**-**f** The sensor was able to identify different person’s pulse rate

Human pulse information holds critical and potential physiological signals, which is highly related to the auxiliary diagnosis of cardiovascular disease [39]. However, the traditional rigid electronics hinders its portable and real-time detection for our better health situation. Here the flexible sensor was conformably fixed on our wrist tightly, as shown in Fig. 5a. The sensor can easily catch the pulse wave of one coauthor (27 year old, 50 kg) and show the heart rate of 69 min^−1^ (Fig. 5b). Furthermore, the characteristic peaks of the pulse waveform, referred to Ps (percussion) wave, T (tidal) wave, and D (diastolic) wave, can be distinguished smoothly. Some signals such as the pulse velocity, time gap, and intensity ratio of the corresponding peaks related to the cardiovascular disease also can be observed, further demonstrating its practical application (Fig. 5c). The pulse waves of two different people were collected to prove its general application (Fig. 5d). Their pulse rate was calculated to be 80 and 69 min^−1^ for the two volunteers, respectively. Besides, the frequency spectrum of them was obtained through fast Fourier transformation (FFT) in Fig. 5e and f, respectively. Both the frequencies were located in the normal range for a healthy person [40]. These above results not only demonstrate the capability of the sensor to reveal the pulse information for a person but also can reflect the individual difference based on the superior sensitivity of our sensor.

**Fig. 6:**
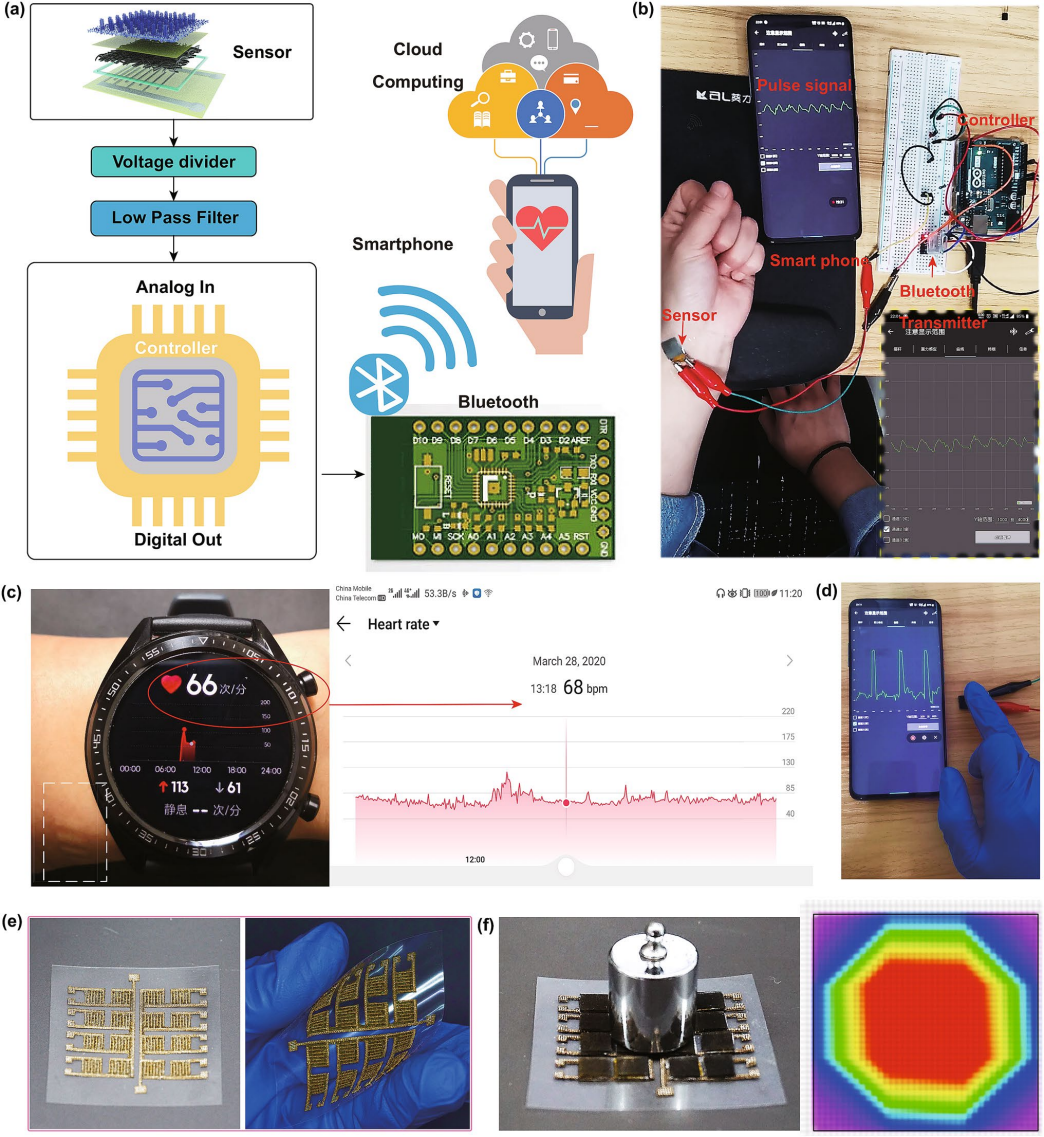
Wireless real-time pulse monitoring of the sensor for healthcare. **a** Device configuration of the wearable sensor system. **b** Practical application of detecting the pulse wave and successfully send the signal to the smartphone. **c** Commercial sports watch showed a similar date value collected by our wearable sensor. **d** Practical application of gentle touching. **e** 4 × 4 sensor array and its corresponding highly flexible circuit diagram. **f** Pressing geometrical area of the weight measured by the sensor array

Furthermore, portable commercial electronics such as the sports watch have been widely developed to make life more convenient and healthier through the real-time data collection of physiological information. However, these rigid characteristics limit the wearer’s comfort and the ability to apply around the clock, so the wireless wearable and comfortable ability is therefore pursued [41]. The flexible sensor therefore was configurated with the voltage divider, low-pass filter, microcontroller, and Bluetooth to send the signal to the mobile phone or cloud computing center (Fig. 6a). As shown in Fig. 6b and Movie S4, the sensor was attached to the body only by thin scotch tape, and then the pulse waves were positively observed through the customized software on the cellphone. The estimated pulse rate of 69 min^−1^ (inset in Fig. 6b) is close to the data obtained by the commercial smartwatch (66–68 min^−1^, Fig. 6c). Still, the commercial electronics present less comfort degree as it needs a very tight bonding on the body to get the true value (as marked by the white circle, Fig. 6c). Also, a continuous gentle touch was precisely revealed on the smartphone (Fig. 6d and Movie S5). Additionally, our sensor was applied to the planter pressure on different location of the subject’s left foot and the max-pressure over 400 kPa was easily detected. Hence, the plantar pressure distribution though integrating this sensor would be in favor for optimizing shoes’ architecture design (Figs. S10 and S15). Based on the above information, it is strongly believed that this kind of sensor holds strong potential in integrating into flexible electronics with comfortability and portable features. Finally, building pressure sensor matrices are necessary to achieve various applications on AI, touch screen panels, and robotic imaging systems [21]. Figure 6e shows a 4 × 4 sensor array and its corresponding highly flexible circuit diagram. The panel can show the pressing geometrical area of the weight through the current intensity, further indicating its large-scale capability for practical application (Fig. 6f).

## Conclusions

In summary, we designed an effective and general approach to fabricate a soft electrode for the high-performance flexible sensor. The fully soft SF materials as the integrated electrode push the sensor’s linear working range to a new level (up to 800 kPa) while remaining an admirable sensitivity (6.417 kPa^−1^) and long-time durability without any obvious decay. Moreover, the sensor showed an ultrafast response time of only 4 ms based on the unique and ordered structure of the soft electrode and the sensing materials. Meanwhile, the waterproof PDMS MPF grafted with CNT anchored on the surface acted as a protective layer of the sensor to further improve its viability in harsh environments such as the raining (CA is approaching 150º), which can also be extended to other flexible electronics. Application of the sensor or sensor matrices configured with the Bluetooth successfully achieved the “communicate-analyze-interpret-decide” function to send the detected wireless signal of pulse rate or applied pressure to the portable smartphone, thereby demonstrating its feasibility and effectiveness as a reliable electronic for health monitoring. This study confirmed that rationally building the electrode was also capable of greatly improving the sensor’s performance instead of merely focusing on the sensing materials. We hoped that this design concept and sensor system could potentially pave the way for more advanced wearable electronics in the future.

## Electronic supplementary material

Below is the link to the electronic supplementary material.

Supplementary material 1 (PDF 1401 kb)

Supplementary material 2 (MP4 153 kb)

Supplementary material 3 (MP4 2595 kb)

Supplementary material 4 (MP4 4949 kb)

Supplementary material 5 (MP4 2600 kb)

Supplementary material 6 (MP4 4338 kb)
