# Supplementary material for: Flexible Waterproof Piezoresistive Pressure Sensors with Wide Linear Working Range Based on Conductive Fabrics
Source: Nanomicro Lett. 2020 Aug 8;12:159. doi: 10.1007/s40820-020-00498-y (PMC7770928; doi:10.1007/s40820-020-00498-y)
Supplement: Supplementary file 1 — Supplementary material 1 (PDF 1401 kb) [file 40820_2020_498_MOESM1_ESM.pdf]

Supporting Information for

Flexible Waterproof Piezoresistive Pressure Sensors with Wide Linear Working Range Based on Conductive Fabrics

Hongcheng Xu[1, 2, †], Libo Gao[1, 2, †, *], Yuejiao Wang[3, 4, †], Ke Cao[1, 2], Xinkang Hu[1, 2], Liang Wang[5], Meng Mu[1, 2], Min Liu[1, 2], Haiyan Zhang[1, 2], Weidong Wang[1, 2, *], Yang Lu[2, 3, 4, *]

[1]School of Mechano-Electronic Engineering, Xidian University, Xi'an 710071, People's Republic of China

[2]CityU-Xidian Joint Laboratory of Micro/Nano-Manufacturing, Xi'an 710071, People's Republic of China

[3]Department of Mechanical Engineering, City University of Hong Kong, Hong Kong SAR, Kowloon 999077, Hong Kong, China

[4]Nano-Manufacturing Laboratory (NML), Shenzhen Research Institute of City University of Hong Kong, Shenzhen 518057, People's Republic of China

[5]Micro-/Nano-technology Research Center, State Key Laboratory for Manufacturing Systems Engineering, Xi'an Jiaotong University, Xi'an 710049, People's Republic of China

[†] Hongcheng Xu, Libo Gao, and Yuejiao Wang contributed equally to this work.

*Corresponding authors. E-mail: lbgao@xidian.edu.cn (Libo Gao); wangwd@mail.xidian.edu.cn (Weidong Wang); yanglu@cityu.edu.hk (Yang Lu)

Supplementary Figures

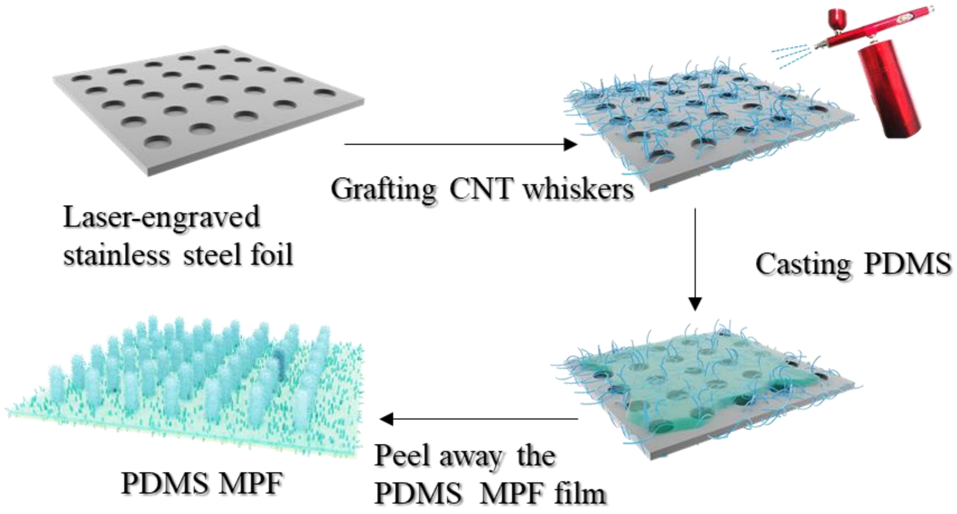

Fig. S1 Experimental process for preparation of PDMS micro pillar film (MPF)

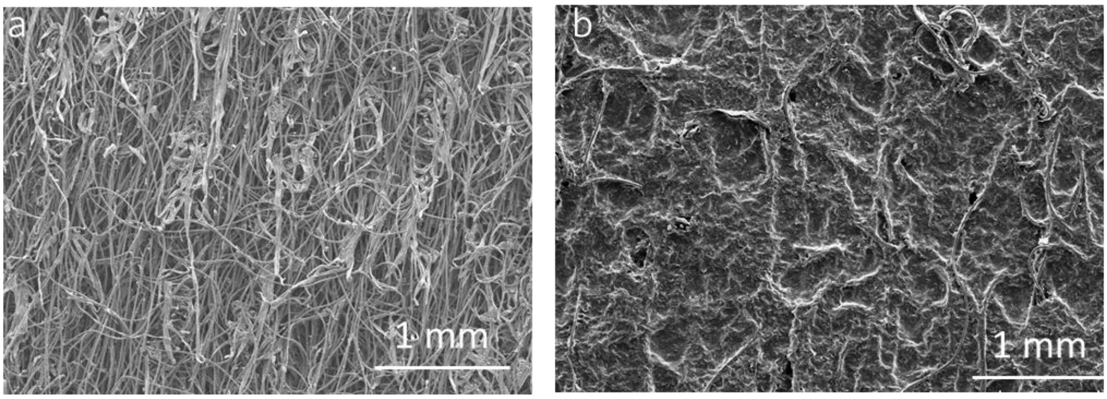

Fig. S2 Scanning electron microscope (SEM) of a non-woven fabrics (nWF) and b Graphite flakes (GFs) coated nWF

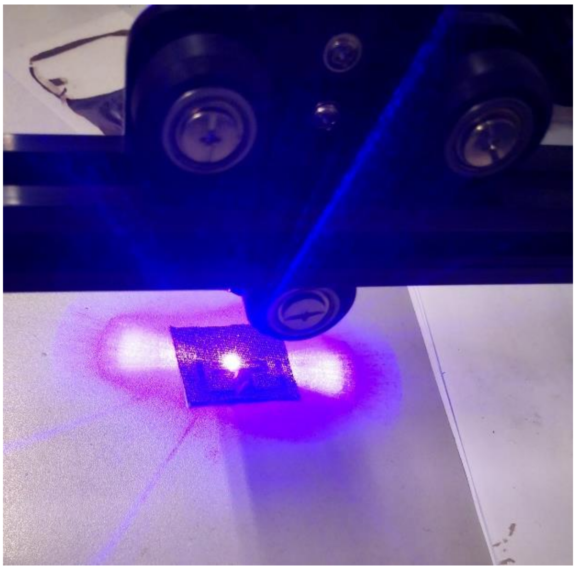

Fig. S3 SF was engraved by the laser

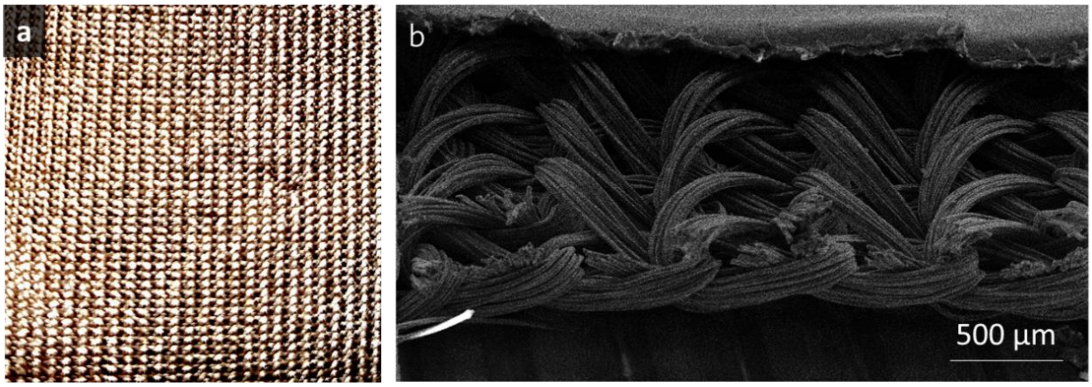

Fig. S4 a Digital optical images of Silver fabrics (SF) on the top view and **b** Side view SEM image of the SF

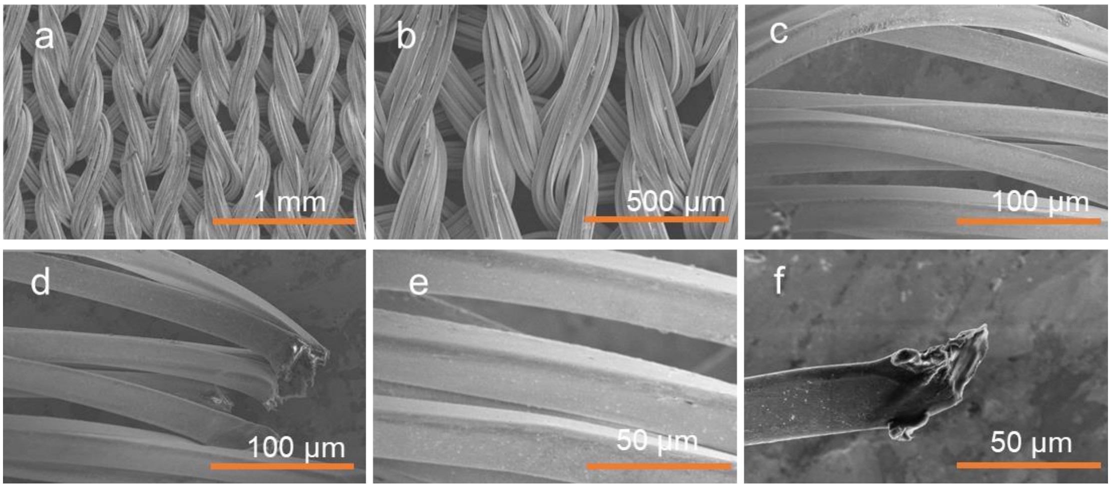

Fig. S5 SEM image of the SF and corresponding silver threads

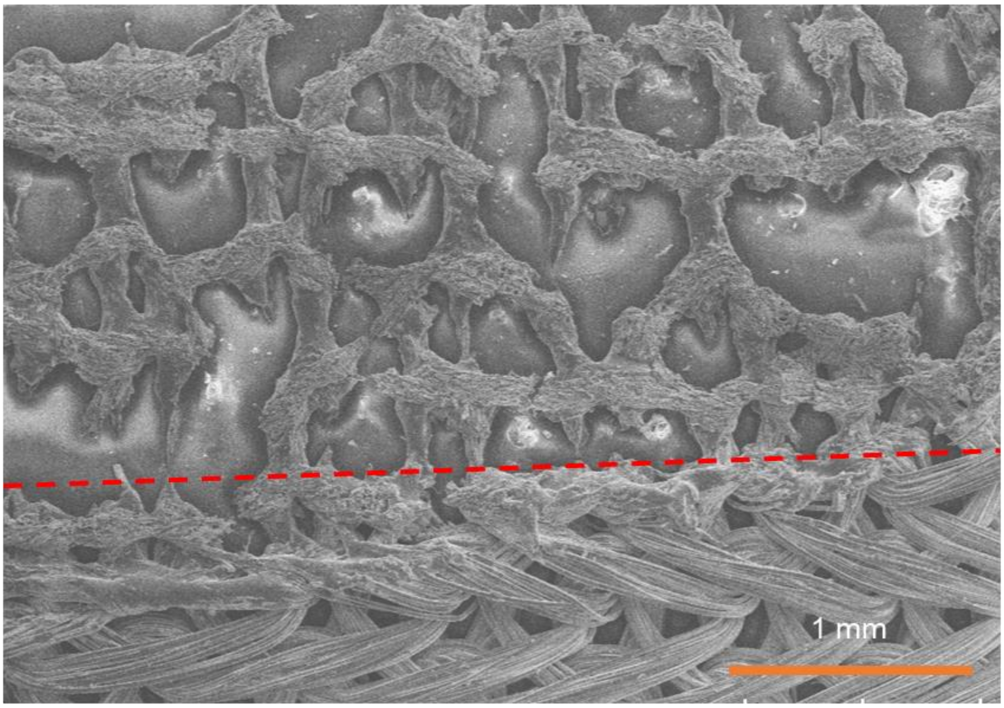

Fig. S6 Optical image and microscopes of the SF

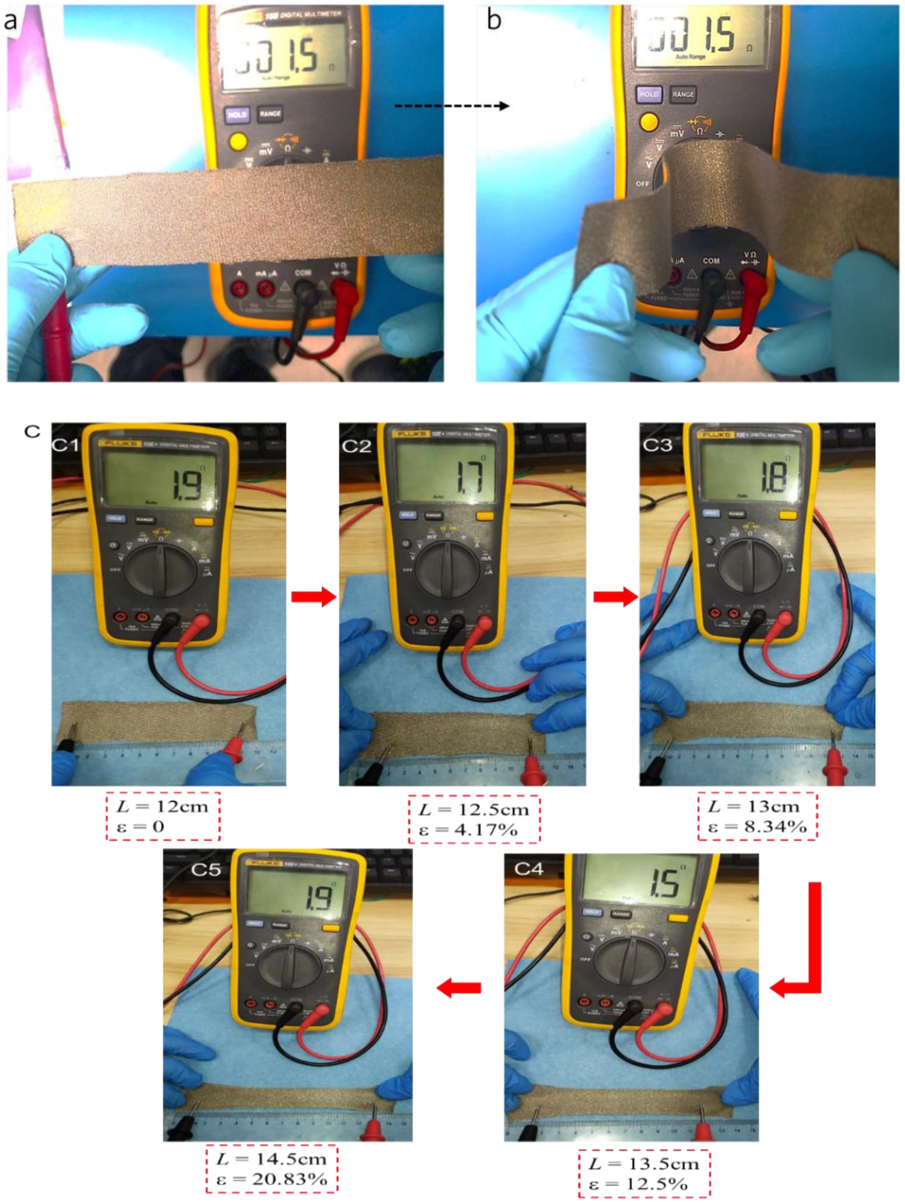

Fig. S7 Resistance of the SF electrode at (**a**) Flat, (**b**) bending, and (**c**) stretching status

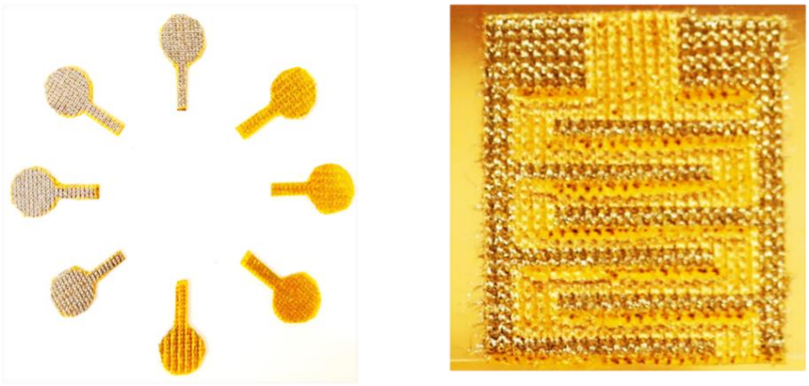

Fig. S8 Various patterns were fabricated by the laser

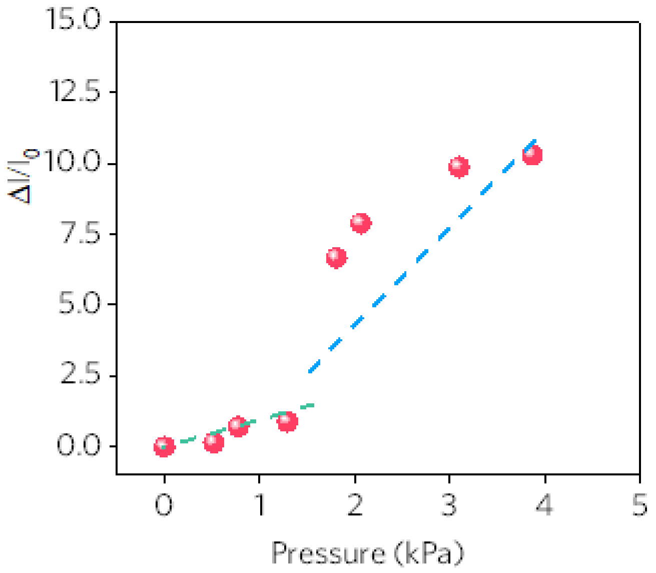

Fig. S9 Current variation at low pressure range

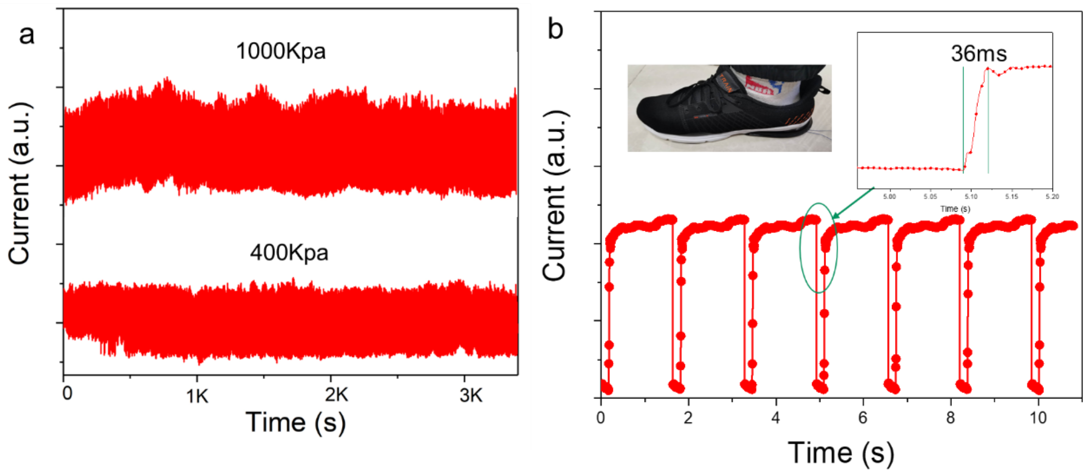

Fig. S10 a Durability test of the sensor under two higher applied pressure of 400 kPa and 1000kpa for 3500s, respectively; **b** practical response time of our sensor under the plantar pressure from one adult male co-author (weight: 75kg)

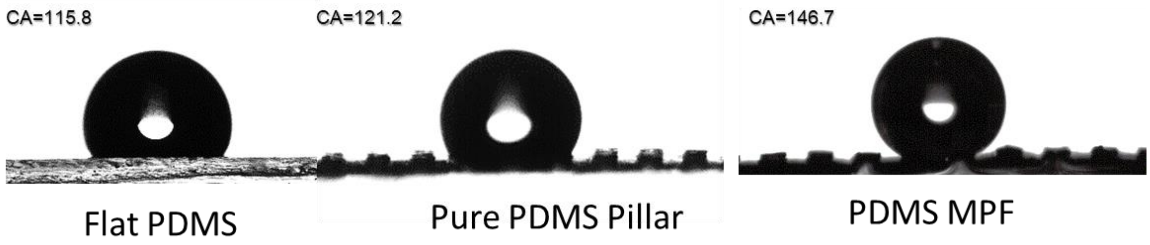

Fig. S11 Water contact angle of various substrate

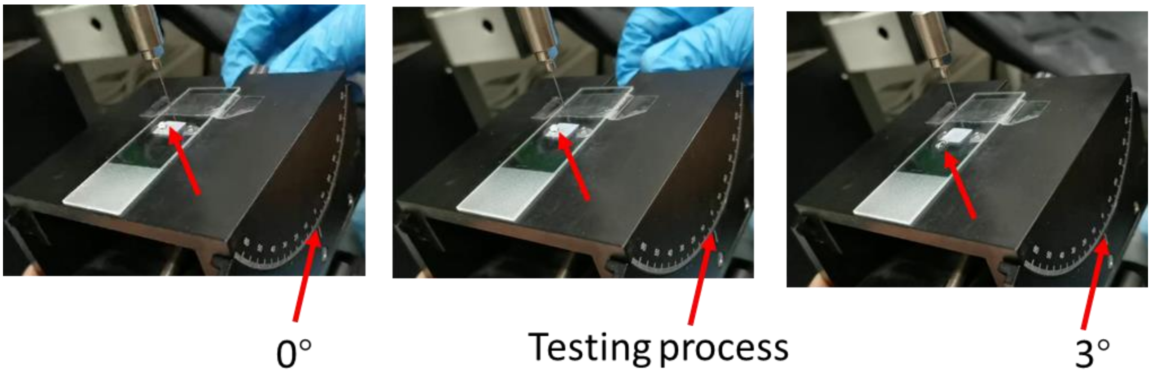

Fig. S12 Roll-off angle testing of water droplet

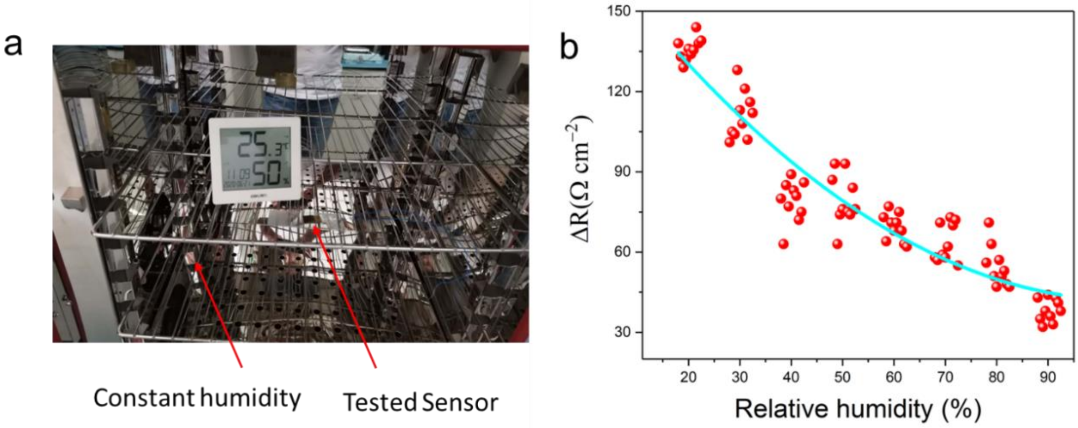

Fig. S13 a testing schematic of the sensor in the constant humidity cabinet; **b** Sensor resistance changes versus different relative humidity

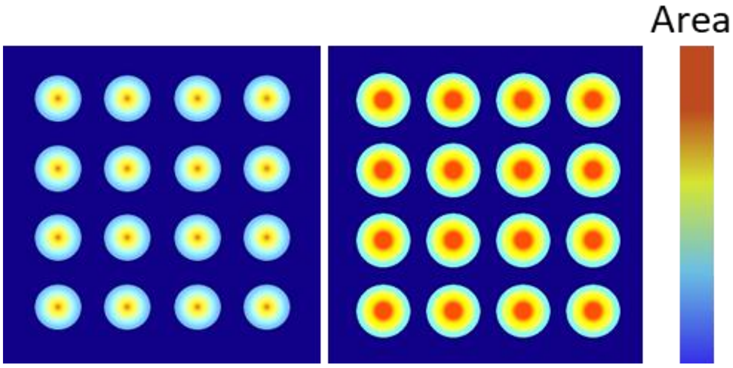

Fig. S14 Contacting area greatly increased with the applied pressure

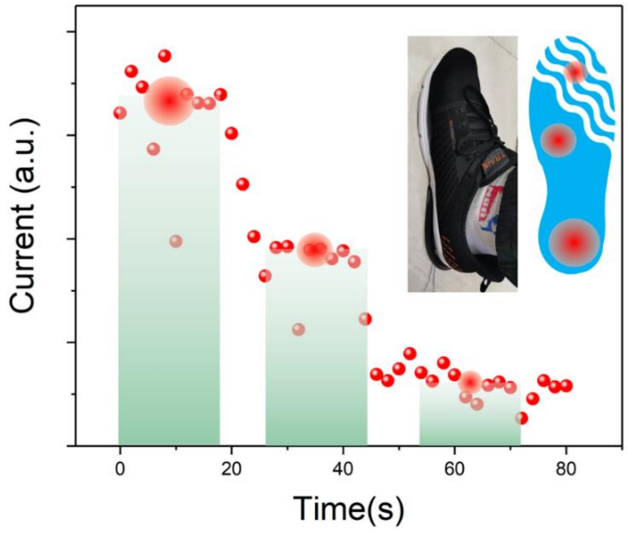

Fig. S15 Practical investigation of our sensor under the plantar higher pressure (over 400 kpa) from one adult male co-author (weight: 75kg)

Supplementary Movies

Movie S1 Water was rapidly removed by the PDMS MPF

Movie S2 Bouncing of the water droplet with 16 times slower

Movie S3 *in situ* transmission electron microscopy (TEM) nano-mechanical test of the graphite flakes (GFs) with 4 times faster

Movie S4 Wearable sensor was able to detect the pulse rate and wirelessly send it to the smart phone

Movie S5 Flexible sensor can catch the gentle touch signal
